# Tuberculin skin test and Quantiferon test agreement and influencing factors in tuberculosis screening of healthcare workers: a systematic review and meta-analysis

**DOI:** 10.1186/s12995-015-0044-y

**Published:** 2015-01-27

**Authors:** Monica Lamberti, Rossella Uccello, Maria Grazia Lourdes Monaco, Mariarosaria Muoio, Daniela Feola, Nicola Sannolo, Albert Nienhaus, Paolo Chiodini

**Affiliations:** Department of Experimental Medicine, Section of Hygiene, Occupational Medicine and Forensic, Medicine, Second University of Naples, Naples, Italy; Center of Excellence for Epidemiology and Health Services Research for Healthcare Professionals (CVcare), Institute for Health Service Research in Dermatology and Nursing, University Medical Center Hamburg-Eppendorf, Hamburg, Germany; Principles of Prevention and Rehabilitation Department (GPR), Institute for Statutory Accident Insurance and Prevention in the Health and Welfare Services (BGW), Hamburg, Germany; Medical Statistics Unit, Second University of Naples, Naples, Italy

**Keywords:** Tuberculosis, Tuberculin skin test, Quantiferon TB Gold, Healthcare workers, Health surveillance, Meta-analysis, Cohen’s k

## Abstract

**Objective:**

A systematic review and meta-analysis was conducted to evaluate the agreement between Tuberculin Skin Test (TST) and Quantiferon (QFT) in screening for tuberculosis (TB) infection among healthcare workers (HCWs) and to estimate associations between TST and QFT agreement and variables of interest, such as *Bacillus Calmette-Guérin* (BCG) vaccination and incidence of TB.

**Methods:**

Cross-sectional and longitudinal studies on HCWs, published in English until October 2013, comparing TST and QFT results, were selected. For each study *Cohen’s κ* value and a 95% confidence interval were calculated. Summary measures and indexes of heterogeneity between studies were calculated.

**Results:**

29 studies were selected comprising a total of 11,434 HCWs. *Cohen’s κ* for agreement between TST and QFT for 24 of them was 0.28 (95% CI 0.22 to 0.35), with the best value in high TB incidence countries and the lowest rate of BCG vaccination.

**Conclusion:**

Currently, there is no gold standard for TB screening and the most-used diagnostic tools show low agreement. For evidence-based health surveillance in HCWs, occupational physicians need to consider a number of factors influencing screening results, such as TB incidence, vaccination status, age and working seniority.

## Introduction

Occupational exposure to biological agents is a major risk for healthcare workers (HCWs) thus in health surveillance much has to be dedicated to infectious disease screening. Tuberculosis (TB) is an ongoing risk in low-income countries due to abandonment of vaccination campaigns, immigrations flows, wide diffusion of primary or secondary immunosuppression, poor efficacy of vaccine currently in use [1,2] so that tuberculosis remains a major public health problem.

According to the World Health Organization (WHO), in 2012 there were an estimated 8.6 million cases of TB (range 8.3–9.0 million) globally, equivalent to 122 cases per 100,000 population. Most of the estimated case numbers in 2012 occurred in Asia (58%) and Africa (27%); smaller proportions occurred in the Eastern Mediterranean region (8%), the European region (4%) and the Americas (3%). One third of the world’s population is estimated to be latently infected with *Mycobacterium tuberculosis*: people with latent TB infection (LTBI) do not show symptoms of TB and are not infectious, but they are at risk of developing active disease and becoming infectious. Several factors increase the risk of progressing from infection to active TB, for example, HIV infection or immunosuppressive treatment, malnutrition, diabetes and alcohol abuse. Preventing active TB by addressing these risk factors as well as proper diagnosis and treatment of LTBI in selected risk groups is thus important for the individual and for public health [3].

It is clear that LTBI diagnosis is mostly based on screening programs that address the general population or occupational categories such as healthcare workers. Actually, there is a lack of gold-standard for LTBI diagnosis: traditionally, TB infection screening is conducted by tuberculin skin testing (TST). Some years ago interferon-gamma release assays (IGRAs) became commercial available. IGRAs are used as a confirmatory test for TST in a two steps procedure or as a replacement of the TST particularly in situations in which the TST is not recommended [4]. TST remains the major tool used around the world for diagnosis of TB infection because of well-established algorithms for test interpretation. In addition TST is easy to use and it has a good cost-effectiveness. Although widely used, TST has limitations; its sensitivity may be reduced by malnutrition, severe TB diseases and immunodeficiency. Decreased TST specificity might occur in settings where *non-tuberculous mycobacteria* (NTM) are prevalent and in populations who have received *Bacillus Calmette-Guérin* (BCG) vaccine post-infancy or via multiple vaccinations [5], although its effect on TST reactions could be modest after 10 years. Additionally, completing the TST requires two healthcare visits, resulting in loss of reading in approximately 10% of cases [6]. This method is affected by inter-observer variability and the positive result does not distinguish recent from remote infection [6].

Most recent national guidelines present IGRAs (especially Quantiferon, QFT) as a new valid tool for diagnosis of latent tuberculosis, also because they are ex-vivo blood-based tests that, in contrast to the TST, can be repeated any number of times without sensitization or boosting, they require only one visit and do not need a baseline two-step protocol [5]. However, reviews have suggested that IGRA performance differs in high versus low TB incidence settings as well as in presence of some risk factors [6]. Moreover, IGRA reproducibility is influenced by several technical factors and immunomodulation [5]; subsequently, appropriate cut-offs and borderline zones need jet to be derived especially for interpreting of IGRA result in serial testing of HCWs in light of an individual’s tuberculosis risk factors [7]. Although a single IGRA is more expensive than the intradermal investigation, the cost-effectiveness analysis depends on epidemiological and individual elements, as explained in several studies that also sought to elaborate specific models [8].

Several recent systematic reviews showed that HCWs are at an increased risk of exposure to *Mycobacterium tuberculosis* [1,2,9,10]. For this reason, periodic screening of HCWs is an important component of TB programs, according to the background TB incidence in the population, resulting in TB as an occupational disease.

In specific working population, such as HCWs, serial testing for TB seems to be more appropriate in order to identify recent infections and to target infected individuals for preventive therapy [8]. Some guidelines from high-income low-incidence countries have not recommended IGRAs for serial testing of HCWs while others state that IGRAs may be used for serial testing of HCWs in place of the TST [11]: according to WHO guidelines [12] IGRAs should not be used in HCW screening programs for low- and middle-income countries (strong recommendation). This indication derives from reversion or conversion rates that reduce IGRA reproducibility.

Until now, there have been various systematic reviews of literature, evaluating prevalence or incidence of latent TB disease among HCWs [9] or IGRA performance [11] for tuberculosis screening in HCWs; agreement between TST and IGRA has been generally evaluated as secondary outcome. A systematic review and meta-analysis had compared the accuracy of Quantiferon TB Gold in Tube and the T-SPOT assays with the TST, but has not considered HCWs and not quantified the agreement between skin testing and IGRA [13].

The present study aims to conduct a systematic review with a meta-analysis of the impact of some factors on the agreement between the two tests (TST and QFT) for TB screening programs in HCWs, measured with *Cohen’s κ* in order to be more rigorous and informative than narrative and systematic reviews [14,15]. The impact of some risk factors on the outcome of those tests has also been examined in order to derive an “evidence-based” protocol for healthcare workers at risk of tuberculosis.

## Methods

### Data sources and searches

A systematic review with meta-analysis, according to the PRISMA (Preferred Reporting Items for Systematic Reviews and Meta-Analyses) statement, was conducted [16].

Original articles were searched through PubMed, Embase, Web of Science and Scopus from 1 January 2004 to17 October 2013, using various combinations, in line with the specific database language, of the terms “workers” AND “tuberculosis” OR “TB infection” or “TB disease” OR “TB” AND “tuberculin skin test” OR “tuberculin skin testing” AND “Quantiferon”. Additional studies were taken by means of reference lists from previous review articles, and citations of relevant original articles were screened.

### Study outcomes and selection

Original articles were evaluated, including cross-sectional and longitudinal studies, meeting all the following criteria: screening of LTBI in HCWs with contemporary TST and QFT, comparison between TST and QFT results, sample vaccination rates, English language. The following studies were excluded: duplicates, case reports or studies on close contacts, editorials, immunological or laboratory studies, NTM studies. Articles about HCWs affected by HIV, chronic rheumatological diseases or inflammatory bowel diseases were also eliminated in order to avoid other influence factors and to obtain a more homogeneous sample.

Epidemiological studies often did not apply to specific medical occupational groups, but instead calculated the risk of infection or disease for the *overall group of healthcare workers* with a highly heterogeneous definition of ‘healthcare worker’ that included both occupational groups with a potentially increased risk and groups without any contact with tuberculosis patients [10]. In studies physicians, nurses, midwives, laboratory personnel, radiographers, medical or nursing students were considered as HCWs; if the authors also included a few contacts or administrative workers, only data on HCWs was extrapolated wherever possible.

Three reviewers (RU, MM, MGLM) independently screened the citations (title and abstract) identified from all sources. Subsequently, full-text articles selected by titles and abstracts were reviewed to identify the final set of eligible studies. Disagreements were resolved by discussion.

### Data extraction

The following characteristics of each study were listed: country, publication and screening years, sample size, type of study, incidence of TB disease in the general population, gender, mean HCW age, working age, type of tuberculin used for testing, BCG vaccination rates (Table 1).

**Table 1:** **Characteristics of studies**

**First author**	**Country**	**n**	**Gender (F/M)**	**Sample age** ^**a**^	**Working seniority** ^**a**^	**Type of study**	**TB incidence 2012** ^**b**^	**Screening year**	**TB real incidence** ^**c**^	**PPD dose**	**Type of PPD** ^**d**^	**BCG vaccination(%)**
Zwerling et al., 2012 [45]	Canada	388	288/100	34,4 (26,9-44,7) y M(range)	5 (2–12) y M(range)	Cross-sectional	4,5	2007-2011	4,8	5 U	PPD-S	36
Khoury et al., 2011 [40]	Minnesota, USA	611	Missing data	/	Start of work 100%	Cross-sectional	3,9	2005-2006	5,3	5 U	PPD-S (as in national guidelines)	82
Fox et al., 2009 [30]	Israel	100	69/31	31 ± 10 y m + SD	TST-: 3,7 ± 6,1 y TST+: 8,7 ± 10,1 y QFT-: 4,6 ± 7,3 y QFT+: 4,2 ± 5,8 y m + SD	Prospective	5,8	2007	6,1	5 U	PPD-S	37
Vinton et al., 2009 [29]	Australia	481	431/50	42 (20–66) y M(range)	/	Cross-sectional	6	2008	6,1	10 U	PPD-S	78
Larcher et al., 2012 [46]	Italy	549	397/152	38,3 (19–64) y m(range)	/	Cross-sectional	2,8	2006-2007	6,7	5 U	PPD-S (as in national guidelines)	38
Nienhaus et al., 2008^e^ [25]	Germany	261	212/49	40 ± 10,4 y m ± SD	/	Cross-sectional	4,5	2006	6,8	2 U	PPD-RT23	38
Soborg et al., 2007^e^ [23]	Denmark	139	115/24	<30 y 17% 30–39 y 30% 40–49 y 33% ≥ 50 y 20%	/	Cross-sectional	6,5	2007	6,9	2 U	PPD-RT23	76
Girardi et al., 2009 [31]	Italy	115	67/48	≤41 y 51% >41 y 49%	/	Cross-sectional	2,8	2004-2005	7,5	5 U	PPD-S	37
Freeman et al., 2012^e^ [47]	New Zealand	325	270/55	18-30 y 49,2% 31–50 y 41,8% 51–60 y 8,9%	/	Cross-sectional	7,6	2007-2008	8,5	5 U	PPD-S (as in national guidelines)	67
Tripodi et al., 2009 [32]	France	148	109/39	20-29 y 28,4% 30–39 y 19.6% 40–49 y 27,7% 50–60 y 24,3%	≤10 y 52,7% > 10 y 47,3%	Cross-sectional	4,3	2006-2007	9,2	2U	PPD-S (Tubertest)	100
Alvarez-Leon et al., 2009 [33]	Spain	134	101/33	33,4 ± 9,4 y m ± SD	10,8 ± 8,2 y m ± SD	Cross-sectional	15	2007	18	2 U	PPD-RT23	35
Casas et al., 2009 [34]	Spain	147	113/34	43,3 (22–63) y M(range)	18,4 (1–43) y m(range)	Cross-sectional	15	2004-2005	19	2 U	PPD-RT23	16
Talebi-Taher et al., 2011 [41]	Iran	200	127/73	34,36 ± 8,26 y m + SD	1-10 y 69,5% 11–20 y 23% 21–30 y 7,5%	Cross-sectional	21	2009-2010	19,5	5 U	PPD SACHIN (Surat) India	100
Hotta et al., 2007^e^ [24]	Japan	207	132/75	20 (18–42) y M(range)	/	Cross-sectional	19	2006	24	3 U	PPD (equivalent to PPD-S)	93
Topic et al., 2009 [35]	Croatia	54	51/3	44 (21–63) y m(range)	/	Cross-sectional	17	2007	25	2 U	PPD-RT23	100
Ozdemir et al., 2010^e^ [38]	Turkey	76	33/43	30,4 ± 5,4 y m + SD	3,9 ± 4,7 y m ± SD	Cross-sectional	24	2005	31	5 U	PPD-S	91
Torres Costa et al., 2011 [42]	Portugal	2884	2068/816	<25 y 10,4% 25–29 y 28,5% 30–39 y 27,4% 40–49 y 18,5% ≥50 y 15,2%	start of work 13,9% <1 y 4,9% 1–5 y 27,3% >5-10 y 16,1% 10–20 y 19,9% ≥20 y 18%	Cohort	24	2007	33	2 U	PPD-RT23	100
Rafiza et al., 2011 [43]	Malaysia	953	839/114	<24 y 30,7% 25–29 y 35,2% 30–34 y 15,6% > 35 y 18,5%	<1 y 14% 1–5 y 47% 6–10 y 17,5% ≥ 11 y 21,5%	Cross-sectional	81	2008-2009	83	2 U	PPD-RT23	100
Choi et al., 2008 [26]	South Korea	80	75/5	28 (23–45) y M(range)	26 (12–240) m M(range)	Cross-sectional	100	2006	90	2 U	PPD-RT23	100
Lee et al., 2009 [36]	South Korea	196	196/0	23,4 ± 1,4 y m + SD	/	Prospective	100	2007	92	2 U	PPD-RT23	93
Lee et al., 2010 [39]	South Korea	82	82/0	28 (22–53) y M(range)	51,5 (0,25-276) m M(range)	Cross-sectional	100	2009-2010	97	2 U	PPD-RT23	100
Moon et al., 2011 [44]	South Korea	173	102/71	32 (22–67) y M(range)	7 (0,2-42) y M(range)	Cross-sectional	100	2010	98	2 U	PPD-RT23	100
Jung et al., 2012 [48]	South Korea	153	48/105	21.9 ± 0.9 y m + SD	/	Cross-sectional	108	2010-2012	102	2 U	PPD-RT23	86
Jo et al., 2013 [50]	South Korea	493	383/110	30,6 ± 6,0 y m + SD	<1 y 8,3% 1–5 y 49,7% 6–10 y 23,1% 11–15 y 13,8% ≥ 15 y 5,1%	Cross-sectional	100	2012	108	2 U	PPD-RT23	81
Whitaker et al., 2013 [51]	Georgia	319	259/60	18-29 y 20% 30–39 y 26% 40–49 y 31% ≥ 50 y 23%	0-4 y 22% 5–14 y 26% 15–24 y 23% ≥ 25 y 29%	Prospective	125	2009-2011	128	5 U	PPD-S	90
Mirtskhulava et al., 2008 [27]	Georgia	265	229/36	42 (18–74) y m(range)	8 (1–42) y M (range)	Cross-sectional	125	2006	160	5 U	PPD-S	78
Lien et al., 2009 [37]	Vietnam	265	197/68	40 (20–58) y M(range)	<2 y 11% ≥2 < 5 y 17,7% ≥5 < 10 y 16,6% ≥10 y 54,7%	Cross-sectional	151	2007	202	5 U	PPD (Pasteur Institute, Vietnam)	33
Pai et al., 2008 [28]	India	719	446/273	22 y M	/	Cohort	249	2004	212	1 U	PPD-RT23	71
He et al., 2012 [49]	Mongolia	917	659/258	18-29 y 22% 30–39 y 23% 40–49 y 35% ≥ 50 y 20%	10 (<1-50) y M(range)	Cross-sectional	223	2010	224	5 U	missing data	34

^a^Data shown through mean (m) + standard deviation (SD) or through mean (m) and range or through Median (M) and range or through percentage (%); / = missing data. Data are shown in months (m) or years (y).

^b^According to WHO data 2012, rates for 100.000 population.

^c^According to WHO data for each screening year, rates for 100.000 population.

^d^According to data reported in each study or cited national guidelines.

^e^Studies not included for the meta-analysis.

According to several national guidelines, different types of purified tuberculin proteins (PPD) were used, PPD RT23 and PPD-S; 5 TU dose of PPD-S (0.1 ml) are accepted as bio-equivalent to 2 TU of PPD RT23 (0.1 ml) [17].

Different types of QFT have been considered, such as in the technological development of IFN-γ release assays over the years: from the first assay, known as Quantiferon-TB, to the last and more specific Quantiferon-TB-Gold in Tube (QFT-GIT), that replaced all other types, which are no longer marketed [18]. In order to provide clear information about IGRA/TST agreement, we considered all of them as “QFT”.

In order to calculate overall agreement between the two main screening tools, data on TST induration (cutoff positivity ≥ 10 mm) and QFT positivity (cutoff positivity 0.35 IU/ml) in HCWs was considered among with the impact of variables of interest on that agreement (vaccination rates and TB incidence). In particular, studies were divided in two groups on the basis of the BCG vaccination rate using different cutoff values because no reference value was available. The incidence of all forms of TB disease in the general population were obtained from the WHO global TB database for each study year, or publication year, if unavailable. Studies were classified in three groups based on TB incidence in each country: low ≤20 cases/100,000, intermediate 21–99 cases/100,000, and high ≥100 cases/100,000. In order to best characterize TB incidence, the screening year(s) was/were identified in each study; studies lasting for two or more years had an incidence rate calculated by mean.

Any discrepancies were resolved by consensus with the help of the team coordinator, thus obtaining an inter-reviewer agreement of 100%.

### Data synthesis and analysis

Study characteristics and results are presented in tables and plots. The primary outcome of the meta-analysis was the *Cohen’s κ.* For each study the κ value and 95% confidence interval (CI) were calculated. Weighted mean effect size was used as a summary measure. Heterogeneity of studies was assessed by using Q statistics and I^2^ [19]. The P value of Q statistics of less than 0.10 was considered significant [20]. I^2^ values of 25%, 50%, and 75% correspond to cutoff points for low, moderate, and high degrees of heterogeneity. If overall heterogeneity was significant, a random-effect model was used, otherwise a fixed-effect model was used. Meta-regression was applied to test difference of study-level covariates. Meta-regression is a regression model that relates the effect to study-level covariates, while assuming additivity of within-study and between-studies components of variance. Restricted maximum likelihood estimators were used to estimate model parameters [21]. A permutation test (using 1,000 re-allocations) was used to assess the true statistical significance of an observed meta-regression finding [22]. Data was analyzed using Stata, version 11.0 (Stata Corp., College Station, TX). All statistical tests were two-sided and p-values <0.05 were regarded as significant.

## Results

### Description of included studies

**Figure 1:**
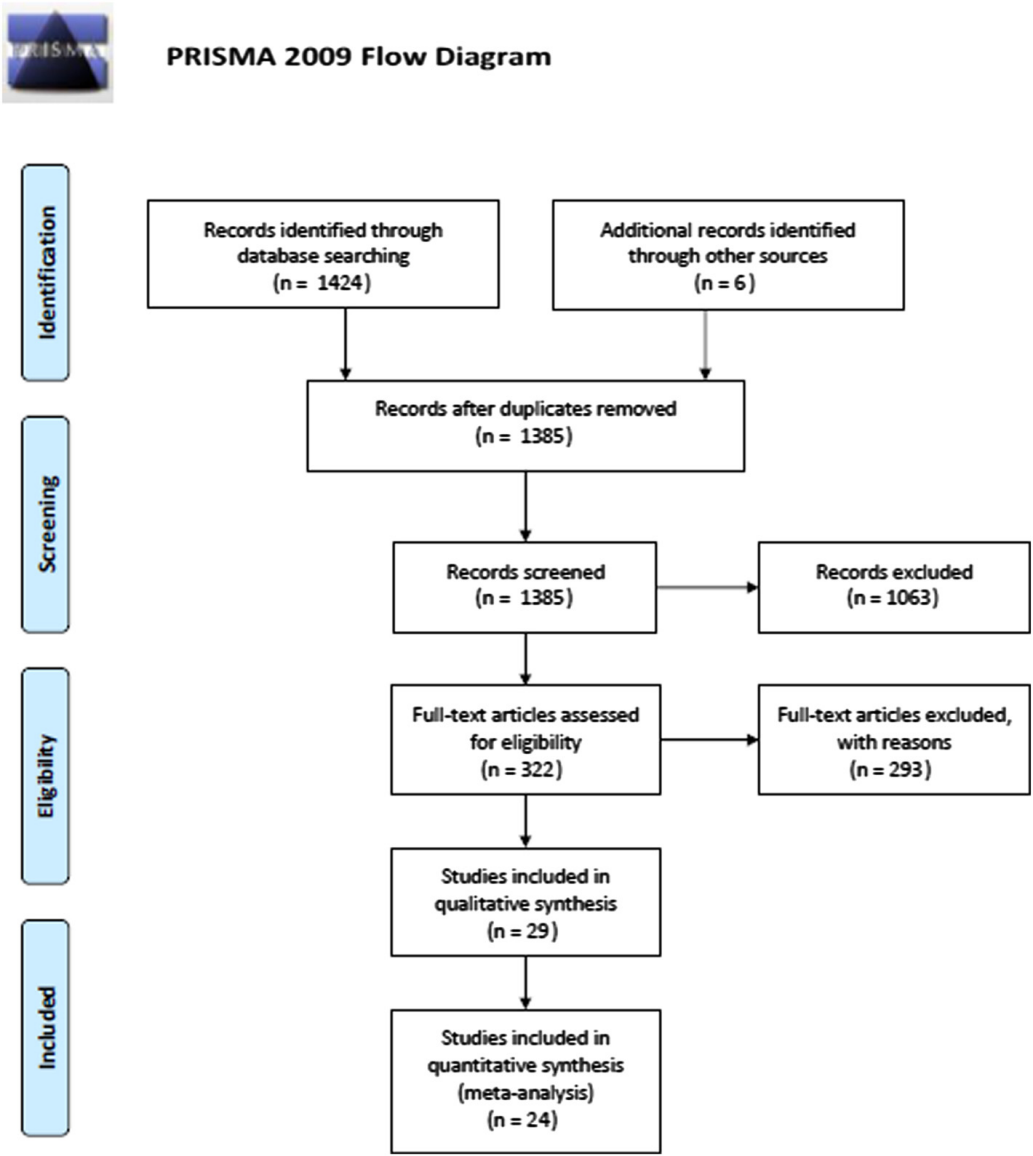
**Flow-chart of studies selection.** From the initial 1430 abstracts, 29 studies met our inclusion criteria. Study sizes ranged from 54 to 2884 HCWs, for a total of 10.314 HCWs across the 29 studies.

Figure 1 shows the flow chart of the studies selection. From the initial 1,430 abstracts, 29 studies [23-51] met our inclusion criteria; 871 studies were discarded as not concerning HCWs, as expected, because a more sensitive than specific search string was preferred in order to obtain as many studies of the health sector as possible. Two hundred and eighty studies were rejected owing to the lack of all results about agreement between TST and QFT or about vaccination status; 13 were rejected for enrolling the same sample of HCWs. Characteristics of the selected studies in this review are shown in Table 1, data are presented by TB incidence. Most studies were cross-sectional (25/29); in prospective studies, baseline information was taken. Study sizes ranged from 54 to 2,884 HCWs, for a total of 11,434 HCWs across the 29 studies. In total, there were 8,098 female and 2,725 male workers; for 611, gender was not indicated [40].

The age mostly represented in the sample was between 30 and 50 or between 18 and 30 when students were included. The overall mean or median age could not be calculated because the sample age was differently reported in the studies as mean, median or percentage.

Working seniority was defined by the number of years or months spent in contact with patients: it played an important role in TB screening results, but in 11 studies data was not shown; in the other 18, this information was heterogeneous (reported through mean, median or percentage). Therefore as with age a mean or median for working seniority could not be calculated; one study carried out a pre-employment evaluation in a healthcare occupational surveillance program [40].

Skin testing was conducted using different types and doses of tuberculin: the dose varied from 1 to 10U according to the national guideline. TST was performed using 2U of RT23 in most studies (14/29) while in 12/29 5U of PPD-S were used.

Among the 29 selected studies, 24 used 10 mm as cutoff for positivity [26-37,39-46,48-51], 2 of them reported 15 mm [24,38], other 2 studies considered 5 mm [25,47] and in only one was the cutoff 12 mm [23]. In order to calculate overall agreement, we considered only studies with 10 mm as cutoff positivity. BCG vaccination rates run from 16 to 100%, with a median of 81%.

**Figure 2:**
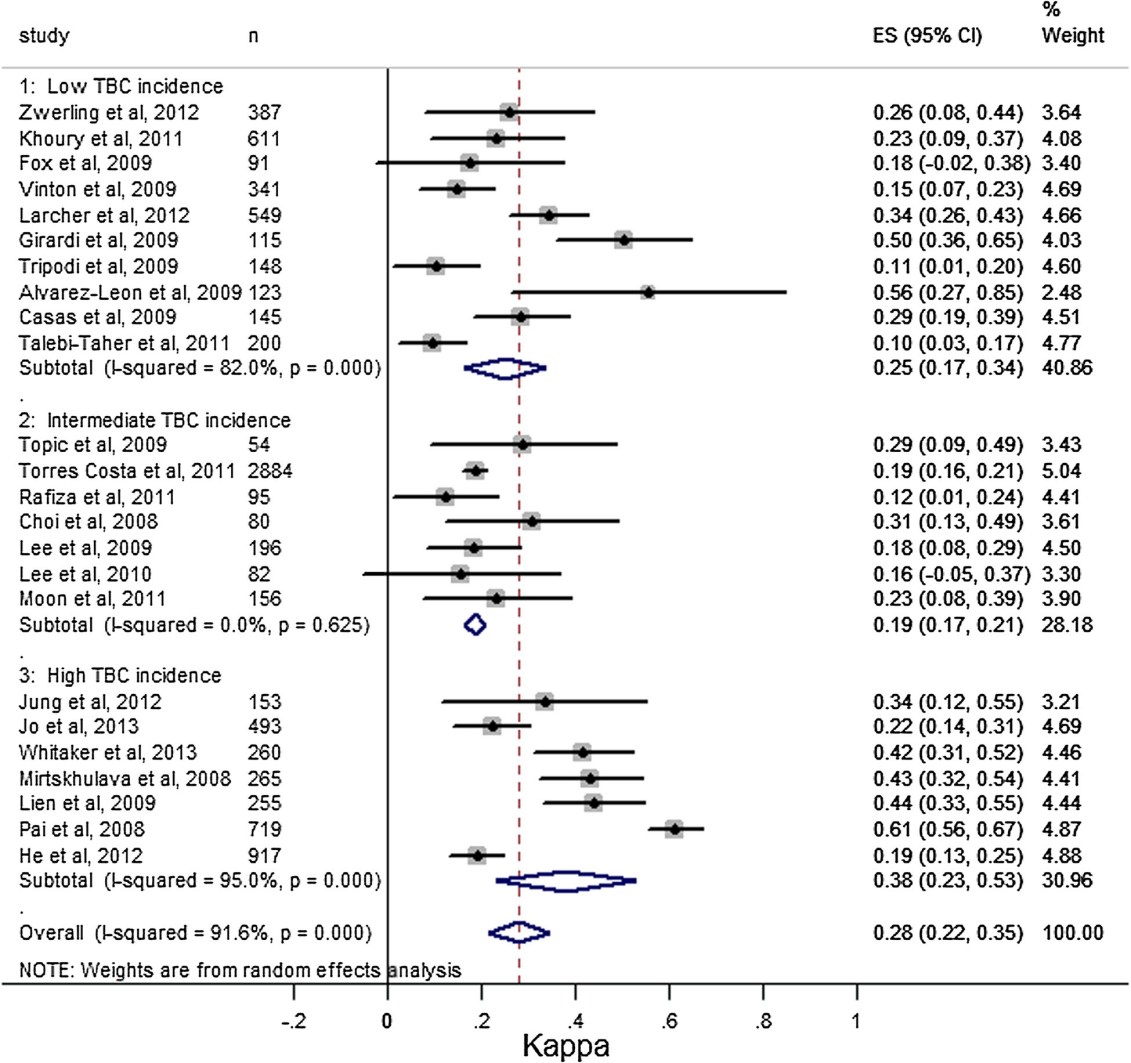
**Meta-analysis of Cohen’s k values for agreement between Tuberculin Skin Test (TST) and Quantiferon TB Gold-In-Tube (QFT) by Tuberculosis (TB) infection incidence classes.** In order to calculate overall agreement, we considered only studies with 10 mm as cutoff positivity. ES: k value.

On the basis of TB incidence identified by WHO information for each year of screening, studies were divided into three groups of incidence: the low TB incidence group was the most represented, being included in 10 studies, followed by the intermediate group with 7 studies and the high group with 7 studies (Figure 2). In a few cases, there was a relevant difference between the two incidence rates [31,32,46,35] owing to a delay in publication.

### Agreement between TST and QFT

Table 2 shows data by TB incidence on subjects with both TST and QFT results, excluding indeterminate QFT results; for this reason the effective sample size was 10,314, or lower than in Table 1.

**Table 2:** **Comparison of TST and QFT results in healthcare workers from different studies**

**First author**	**Sample size (n)**	**TST+ (n)**	**QFT+ (n)**	**TST+/QFT+ (n)**	**TST+/QFT- (n)**	**TST-/QFT+ (n)**	**TST-/QFT- (n)**
Zwerling et al., 2012 [45]	387^a^	22	24	7	15	17	348
Khoury et al., 2011 [40]	611	50	12	8	42	4	557
Fox et al., 2009 [30]	91^a^	31	17	9	22	8	52
Vinton et al., 2009 [29]	341^a^	114	21	16	98	5	222
Larcher et al., 2012 [46]	549	160	81	57	103	24	365
Nienhaus et al., 2008^b^ [25]	261	63	25	15	48	10	188
Soborg et al., 2007^b^ [23]	139	47	2	2	45	0	92
Girardi et al., 2009 [31]	115	61	40	36	25	4	50
Freeman et al., 2012^b^ [47]	317^a^	67	28	19	48	9	241
Tripodi et al., 2009 [32]	148	97	51	23	74	5	46
Alvarez-Leon et al., 2009 [33]	123^a^	9	8	5	4	3	111
Casas et al., 2009 [34]	145^a^	101	43	42	59	1	43
Talebi-Taher et al., 2011 [41]	200	105	17	14	91	3	92
Hotta et al., 2007^b^ [24]	202^a^	120	3	3	117	0	82
Topic et al., 2009 [35]	54	34	17	15	19	2	18
Ozdemir et al., 2010^b^ [38]	76	41	65	39	2	26	9
Torres Costa et al., 2011 [42]	2,884	2,102	953	850	1252	103	679
Rafiza et al., 2011 [43]	95a	56	13	11	45	2	37
Choi et al., 2008 [26]	80	36	16	13	23	3	41
Lee et al., 2009 [36]	196	93	28	22	71	6	97
Lee et al., 2010 [39]	82	31	19	10	21	9	42
Moon et al., 2011 [44]	156^a^	52	32	18	34	14	90
Jung et al., 2012 [48]	153	23	8	6	17	2	128
Jo et al., 2013 [50]	493	181	85	54	127	31	281
Whitaker et al., 2013 [51]	260^a^	145	112	90	55	22	93
Mirtskhulava et al., 2008 [27]	265	177	159	133	44	26	62
Lien et al., 2009 [37]	255^a^	163	135	114	49	21	71
Pai et al., 2008 [28]	719	298	288	226	72	62	359
He et al., 2012 [49]	917	439	638	350	89	288	190
*Total*	*10,314*	*4,918*	*2,940*	*2,207*	*2,711*	*710*	*4,686*

^a^we consider only subjects with both TST and QFT results, excluding undeterminate quantiferon results.

^b^Studies not included in the meta-analysis.

Screening results were reported as positive TST alone, positive QFT alone and crossed of TST and QFT. Out of the 10,314 tests performed, TST and QFT agreed for 6,893 of them and failed to do so for 3,421. TST positive QFT negative discordance occurred about four times more often than TST negative QFT positive discordance [2,711 (26.3%) versus 710 (6.9%)].

In order to evaluate TST and QFT agreement, a statistical analysis was conducted using *Cohen’s κ* in each study. However, only for 24/29 studies, which used a TST positivity cutoff at 10 mm, overall agreement was calculated. *Κ* values appeared in a wide range from 0.10 to 0.61, with a significant heterogeneity (p < 0.0001, I^2^ = 91.6%). Overall *κ* value, estimated using the random effect model, was 0.28 (95% CI 0.22 to 0.35), which is quite low reflecting that almost one third of TST and QFT results were discordant (Table 2).

### Association between κ value and variables of interest

Studies were classified according to type and dose of PPD. No significant difference (p = 0.717) was found between studies with <5 TU (κ = 0.27, 95% CI 0.17 to 0.37) versus ≥5 TU (κ = 0.29, 95% CI 0.21 to 0.38).

According to TB incidence classification, TST and QFT agreement was calculated with *Cohen’s κ* resulting in 0.25 (95% CI 0.17 to 0.34) in the low incidence group, 0.19 (95% CI 0.17 to 0.21) in the intermediate, and 0.38 (95% CI 0.23 to 0.53) in the high group. The best agreement was observed in the high incidence group, while the worst was seen in the intermediate one, with the highest rate of vaccination; comparing the three *κ* figures there was a significant difference between the intermediate and the high incidence group (p = 0.041).

**Figure 3:**
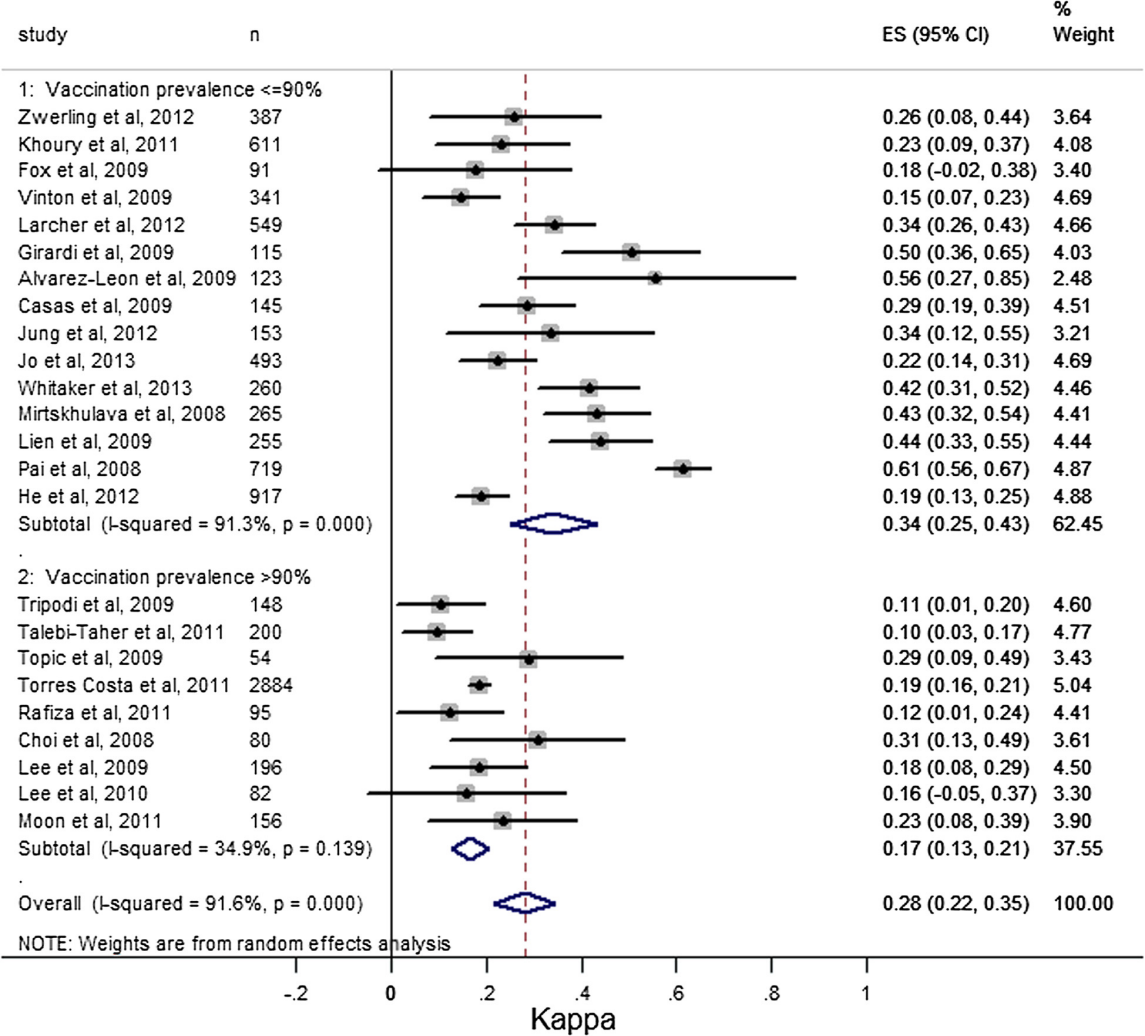
**Meta-analysis of Cohen’s k values for agreement between Tuberculin Skin Test (TST) and Quantiferon TB Gold-In-Tube (QFT) by Bacillus Calmette-Guérin (BCG) vaccination groups.** ES: k value.

Furthermore, studies were divided in two groups (lower and higher vaccination rate) to best elucidate BCG vaccination impact on agreement; considering a 90% cutoff value as a statistically significant difference (p = 0.013) was found, with an agreement of 0.34 (95% CI 0.25 to 0.43) in the lower rate group (15 studies), and 0.17 (95% CI 0.13 to 0.21) in the higher group (9 studies) (Figure 3).

## Discussion

This meta-analysis had the aim of analyzing screening tools for diagnosis of TB infection among HCWs and examining the impact of some risk factors on the outcome of those tests in order to derive an “evidence-based” protocol for screening of healthcare workers at risk of tuberculosis. As already highlighted [11], overall agreement between TST and QFT was quite low. This result can be related to the different immunological targets of the two tests, so that any immunological dysfunction can variously influence their respective results. Moreover, QFT is often characterized by fluctuating results so that its reproducibility is unclear [52]; on the other hand, TST has an inter-observer variability.

TST and QFT also differ in specificity and sensitivity. A lower rate of positivity in the QFT can be explained by a higher specificity of QFT than TST that could come from the intrinsic difference in the methods used by the two tests. QFT uses antigens showing higher specificity to *Mycobacterium tuberculosis* and to only a limited number of NTM, in contrast with the tuberculin used in TST, which represents a mixture of more than 200 nonspecific antigens shared with NTM and with the strains developed from *Mycobacterium bovis* used for BCG vaccination [53].

An important feature of the present systematic review was the high heterogeneity of the studies chosen owing to different impact of each variable (vaccination rates, incidence of TB in each country, age, working seniority, induration diameter cutoff and type of PPD) on the TST/QFT agreement. Again, this variability could be considered as a strength of the study because it offered an opportunity to best elucidate the agreement, taking these factors into account.

BGC vaccination status and incidence of TB, influencing TST and QFT agreement at the same time, could not be valued separately. BCG vaccination reduced the agreement, influencing the TST positivity (rather than QFT positivity) and increasing the risk of a false positive result, especially in recently vaccinated subjects. Although some studies [29,45,47,34] showed a QFT positivity amount lower than the TST positivity one, in the BCG vaccinated group, this result could not be explained with a cross-reaction between vaccination and QFT antigens, but with a TB infection among vaccinated subjects. In the high TB incidence group, a vaccination status lower than 90% was found and, at the same time, the higher observed agreement, although two studies in particular contributed to the decrease of the agreement [49,50]; in detail, some authors found high rates of TST-/QFT+ [49] and others found high rates of TST+/QFT- [50]. In the first case, the result could be explained by a high TB risk ward and a history of TB infection for some subjects; in the other study, false positive results can be explained by re-vaccination. Some authors also affirmed that repeated vaccination influenced quantitative TST positivity but decreased a probability of positive QFT in the case of three or more repeated doses [42]. Moreover, in the low TB incidence group, studies with higher agreement were characterized by the lower rate of vaccination.

Increasing age of HCWs is correlated with concordant TST and QFT positive results [33,34]; Discordant QFT positive TST negative results were associated with an age of over 40 [46,50] or over 50 [49,42] and anyhow this association increased each year [39], although data was not statistically elaborated due to heterogeneity of presentation.

In consideration of working seniority and TST/QFT results, most studies [27,44,42,49] found an association between increasing working years and positivity of both tests; this information cannot be accounted for alone but it has to be contextualized in the risk evaluation at each worksite (high or low TB risk ward). However, in this review there was no statistical analysis of overall age or working seniority possible because of heterogeneity of data presentation or missing information.

Among the studies considering 10 mm and 15 mm as cutoff diameters, three showed better agreement when shifting TST cutoff from 10 mm to 15 mm [29,35,43]; instead, in five studies there was no meaningful difference [33,24,26,44,27].

Some authors affirmed that the type of tuberculin could play a significant effect on skin response: both vaccinated and unvaccinated subjects receiving RT23 2 TU or 1TU were more likely to have a positive result than those receiving 5 TU PPD [54]. Others [55] considered that TST induration size was larger with PPD-S than with PPD RT23 at 48, 72 and 96 hours, resulting in a statistically lower number of false negatives with PPD-S than with PPD RT23. Despite this evidence, no significant difference was found between various types and doses of tuberculin in the chosen studies, confirming bio-equivalence of RT23 and PPD-S [17].

### Study limitations and strength

This systematic review has several strengths. A more sensible than specific search string was elaborated, using multiple databases. Three reviewers (RU, MM, MGLM) independently assessed eligible articles for inclusion. Selection criteria were quite restrictive, so that information obtained was as comparable as possible in order to realize a meta-analysis of overall agreement between the two main screening tools and the influence of some factors (BCG vaccination, TB incidence). However, this was not possible for other variables of interest (age and working seniority) owing to heterogeneity in study design, outcomes and data presentation, despite the limited selection. Still, different national guidelines contribute different study characteristics, particularly on TST procedures and vaccine indications. Lastly, there was a lack of evidence at the highest level of hierarchy on reference standards: a majority of the studies included were cross-sectional. Our study would have appeared more relevant if we had considered longitudinal studies and TST/QFT agreement in serial testing, analyzing all factors that could impact on reversion and conversion. Nevertheless, longitudinal studies did not allow us to analyze both tests in each measurement because it is not always appropriate to repeat both TST and QFT in every HCW.

## Conclusion

Screening for TB infection is a major objective of health surveillance programs. Nowadays, even if there is no gold standard, the most-used diagnostic tools are TST and IGRAs (such as QFT) that show a low agreement and are also influenced by few variables that partially justify their variability alone.

Choosing the proper protocol is a prerogative of the occupational physician, who needs to know about TB and BCG vaccination incidence in the general population and the immunological status and risk factors for each individual worker. TST remains the first-step exam, especially when a higher agreement can be expected, i.e. when there is a low prevalence of vaccination or a high incidence of TB infection. Indeed, QFT is helpful in cases of a higher prevalence of vaccination. Further studies with a unique protocol of health surveillance carried out in variously burdened countries will best clarify the role of TST and QFT for HCW screening.

## References

[1] Baussano I, Nunn P, Williams B, Pivetta E, Bugiani M, Scano F (2011). Tuberculosis among health care workers. Emerg Infect Dis.

[2] Menzies D, Joshi R, Pai M (2007). Risk of tuberculosis infection and disease associated with work in health care settings. Int J Tuberc Lung Dis.

[3] World Health Organization (WHO): Global tuberculosis Report 2013 [http://apps.who.int/iris/bitstream/10665/91355/1/9789241564656_eng.pdf]

[4] Centers for Disease Control and Prevention (CDC) (2005). Guidelines for Preventing the Transmission of Mycobacterium tuberculosis in Health-Care Settings. MMWR.

[5] Pollock NR, McAdam AJ, Pai M, Nardell EA, Bernardo J, Banaei N (2014). Interferon γ-release assays for diagnosis of latent tuberculosis in healthcare workers in low-incidence settings:pros and cons. Clin Chem.

[6] Trajman A, Steffen RE, Menzies D (2013). Interferon-Gamma Release Assays versus Tuberculin Skin Testing for the Diagnosis of Latent Tuberculosis Infection: An Overview of the Evidence. Pulm Med.

[7] Pai M, Kik SV, Banaei N (2014). Occupational screening for tuberculosis. A testing time for interferon-γ release assays. Ann Am Thorac Soc.

[8] Pai M, Denkinger CM, Kik SV, Rangaka MX, Zwerling A, Oxlade O (2014). Gamma interferon release assays for detection of Mycobacterium tuberculosis infection. Clin Microbiol Rev.

[9] Joshi R, Reingold AL, Menzies D, Pai M (2006). Tuberculosis among health-care workers in low- and middle-income countries: a systematic review. PLoS Med.

[10] Seidler A, Nienhaus A, Diel R (2005). Review of epidemiological studies on the occupational risk of tuberculosis in low-incidence areas. Respiration.

[11] Zwerling A, van den Hof S, Scholten J, Cobelens F, Menzies D, Pai M (2012). Interferon-gamma release assays for tuberculosis screening of healthcare workers: a systematic review. Thorax.

[12] World Health Organization (WHO): Use of tuberculosis interferon-gamma release assays (IGRAs) in low- and middle-income countries: Policy Statement. 2011, WHO/HTM/TB/2011.18. http://www.who.int/tb/laboratory/policy_statements/en. 26269875

[13] Diel R, Goletti D, Ferrara G, Bothamley G, Cirillo D, Kampmann B (2011). Interferon-γ release assays for the diagnosis of latent Mycobacterium tuberculosis infection: a systematic review and meta-analysis. Eur Respir J.

[14] Sun S (2011). Meta-analysis of Cohen’s kappa. Health Serv Outcomes Res Method.

[15] Trikalinos TA, Balion CM (2012). Chapter 9: Options for Summarizing Medical Test Performance in the Absence of a “Gold Standard”. J Gen Intern Med.

[16] Moher D, Liberati A, Tetzlaff J, Altman DG, PRISMA Group (2009). Preferred reporting items for systematic reviews and meta-analyses: the PRISMA statement. J Clin Epidemiol.

[17] Comstock GW, Edwards LB, Philip RN, Winn WA (1964). A comparison in the United States of America of two tuberculins, PPD-S and RT23. Bull WHO.

[18] Centers for Disease Control and Prevention (CDC) (2005). Guidelines for Using the QuantiFERON®-TB Gold Test for Detecting Mycobacterium tuberculosis Infection, United States. MMWR.

[19] Deeks JJAD, Bradburn MJ, Egger MD-SG, Altman DG (2001). Statistical methods for examining heterogeneity and combining results from several studies in meta-analysis. Systematic Reviews in Health Care.

[20] Van Houwelingen HC, Arends LR, Stijnen T (2002). Advanced methods in meta-analysis: multivariate approach and meta-regression. Stat Med.

[21] Thompson SG, Sharp SJ (1999). Explaining heterogeneity in meta-analysis: a comparison of methods. Stat Med.

[22] Higgins JPT, Thompson SG (2004). Controlling the risk of spurious findings from meta-regression. Stat Med.

[23] Soborg B, Andersen AB, Larsen HK, Weldingh K, Andersen P, Kofoed K (2007). Detecting a low prevalence of latent tuberculosis among health care workers in Denmark detected by M. tuberculosis specific IFN-gamma whole-blood test. Scand J Infect Dis.

[24] Hotta K, Ogura T, Nishii K, Kodani T, Onishi M, Shimizu Y (2007). Whole blood interferon-gamma assay for baseline tuberculosis screening among Japanese healthcare students. PLoS One.

[25] Nienhaus A, Schablon A, Bâcle CL, Siano B, Diel R (2008). Evaluation of the interferon-gamma release assay in healthcare workers. Int Arch Occup Environ Health.

[26] Choi JC, Shin JW, Kim JY, Park IW, Choi BW, Lee MK (2008). The effect of previous tuberculin skin test on the follow-up examination of whole-blood interferon-gamma assay in the screening for latent tuberculosis infection. Chest.

[27] Mirtskhulava V, Kempker R, Shields KL, Leonard MK, Tsertsvadze T, del Rio C (2008). Prevalence and risk factors for latent tuberculosis infection among health care workers in Georgia. Int J Tuberc Lung Dis.

[28] Pai M, Dendukuri N, Wang L, Joshi R, Kalantri S, Rieder HL (2008). Improving the estimation of tuberculosis infection prevalence using T-cell-based assay and mixture models. Int J Tuberc Lung Dis.

[29] Vinton P, Mihrshahi S, Johnson P, Jenkin GA, Jolley D, Biggs BA (2009). Comparison of QuantiFERON-TB Gold In-Tube Test and tuberculin skin test for identification of latent Mycobacterium tuberculosis infection in healthcare staff and association between positive test results and known risk factors for infection. Infect Control Hosp Epidemiol.

[30] Fox BD, Kramer MR, Mor Z, Preiss R, Rusanov V, Fuks L (2009). The QuantiFERON-TB-GOLD assay for tuberculosis screening in healthcare workers: a cost-comparison analysis. Lung.

[31] Girardi E, Angeletti C, Puro V, Sorrentino R, Magnavita N, Vincenti D (2009). Estimating diagnostic accuracy of tests for latent tuberculosis infection without a gold standard among healthcare workers. Euro Surveill.

[32] Tripodi D, Brunet-Courtois B, Nael V, Audrain M, Chailleux E, Germaud P (2009). Evaluation of the tuberculin skin test and the interferon-γ release assay for TB screening in French healthcare workers. J Occup Med Toxicol.

[33] Alvarez-León EE, Espinosa-Vega E, Santana-Rodríguez E, Molina-Cabrillana JM, Pérez-Arellano JL, Caminero JA (2009). Screening for tuberculosis infection in spanish healthcare workers: Comparison of the QuantiFERON-TB gold in-tube test with the tuberculin skin test. Infect Control Hosp Epidemiol.

[34] Casas I, Latorre I, Esteve M, Ruiz-Manzano J, Rodriguez D, Prat C (2009). Evaluation of interferon-gamma release assays in the diagnosis of recent tuberculosis infection in health care workers. PLoS One.

[35] Topić RZ, Dodig S, Zoricić-Letoja I (2009). Interferon-gamma and immunoglobulins in latent tuberculosis infection. Arch Med Res.

[36] Lee K, Han MK, Choi HR, Choi CM, Oh YM, Lee SD (2009). Annual incidence of latent tuberculosis infection among newly employed nurses at a tertiary care university hospital. Infect Control Hosp Epidemiol.

[37] Lien LT, Hang NT, Kobayashi N, Yanai H, Toyota E, Sakurada S (2009). Prevalence and risk factors for tuberculosis infection among hospital workers in Hanoi. Viet Nam. PLoS One.

[38] Ozdemir D, Annakkaya AN, Tarhan G, Sencan I, Cesur S, Balbay O (2007). Comparison of the tuberculin skin test and the quantiferon test for latent Mycobacterium tuberculosis infections in health care workers in Turkey. Jpn J Infect Dis.

[39] Jong Lee K, Ae Kang Y, Mi Kim Y, Cho SN, Wook Moon J, Suk Park M (2010). Screening for latent tuberculosis infection in South Korean healthcare workers using a tuberculin skin test and whole blood interferon-gamma assay. Scand J Infect Dis.

[40] Khoury NZ, Binnicker MJ, Wengenack NL, Aksamit TR, Buchta WG, Molella RG (2011). Preemployment screening for tuberculosis in a large health care setting: comparison of the tuberculin skin test and a whole-blood interferon-γ release assay. J Occup Environ Med.

[41] Talebi-Taher M, Javad-Moosavi SA, Entezari AH, Shekarabi M, Parhizkar B (2011). Comparing the performance of QuantiFERON TB Gold and Mantoux test in detecting latent tuberculosis infection among Iranian heath care workers. Int J Occup Med Environ Health.

[42] Torres Costa J, Silva R, Ringshausen FC, Nienhaus A (2011). Screening for tuberculosis and prediction of disease in Portuguese healthcare workers. J Occup Med Toxicol.

[43] Rafiza S, Rampal KG, Tahir A (2011). Prevalence and risk factors of latent tuberculosis infection among health care workers in Malaysia. BMC Infect Dis.

[44] Moon HW, Kim H, Hur M, Yun YM, Lee A (2011). Latent tuberculosis infection screening for laboratory personnel using interferon-γ release assay and tuberculin skin test in Korea: an intermediate incidence setting. J Clin Lab Anal.

[45] Zwerling A, Cojocariu M, McIntosh F, Pietrangelo F, Behr MA, Schwartzman K (2012). TB screening in Canadian health care workers using interferon-gamma release assays. PLoS One.

[46] Larcher C, Frizzera E, Pretto P, Lang M, Sonnleitner N, Huemer HP (2012). Immunosurveillance for Mycobacterium tuberculosis of health care personnel in a third level care hospital. Med Lav.

[47] Freeman JT, Marshall RJ, Newton S, Austin P, Taylor S, Chew TC (2012). Screening for Mycobacterium tuberculosis infection among healthcare workers in New Zealand: prospective comparison between the tuberculin skin test and the QuantiFERON-TB Gold In-Tube assay. N Z Med J.

[48] da Jung H, Jo KW, Shim TS (2012). Prevalence of Latent Tuberculosis Infection among Medical Students in South Korea. Tuberc Respir Dis (Seoul).

[49] He GX, Wang LX, Chai SJ, Klena JD, Cheng SM, Ren YL (2012). Risk factors associated with tuberculosis infection among health care workers in Inner Mongolia, China. Int J Tuberc Lung Dis.

[50] Jo KW, Hong Y, Park JS, Bae IG, Eom JS, Lee SR (2013). Prevalence of Latent Tuberculosis Infection among Health Care Workers in South Korea: A Multicenter Study. Tuberc Respir Dis (Seoul).

[51] Whitaker J, Mirtskhulava V, Kipiani M, Harris DA, Tabagari N, Kempker RR (2013). Prevalence and incidence of latent tuberculosis infection in georgian healthcare workers. PLoS One.

[52] Zhao X, Mazlagic D, Flynn EA, Hernandez H, Abbott CL (2009). Is the QuantiFERON-TB blood assay a good replacement for the tuberculin skin test in tuberculosis screening? a pilot study at Berkshire Medical Center. Am J Clin Pathol.

[53] Nahid P, Pai M, Hopewell PC (2006). Advances in the diagnosis and treatment of tuberculosis. Proc Am Thorac Soc.

[54] Wang L, Turner MO, Elwood RK, Schulzer M, FitzGerald JM (2002). A meta-analysis of the effect of Bacille Calmette-Guérin vaccination on tuberculin skin test measurements. Thorax.

[55] Morán-Mendoza O, Tello-Zavala MC, Rivera-Camarillo M, Ríos-Meza Y (2013). Comparison of different methods and times for reading the tuberculin skin test. Int J Tuberc Lung Dis.

